# Network structure induced bias in estimates of intrinsic generation times

**DOI:** 10.1371/journal.pcbi.1014239

**Published:** 2026-05-12

**Authors:** Pratyush K. Kollepara, Chiara Poletto, Joel C. Miller

**Affiliations:** 1 Department of Mathematical and Physical Sciences, La Trobe University, Melbourne, Australia; 2 Department of Molecular Medicine, University of Padova, Padova, Italy; 3 Australian Centre for AI in Medical Innovation, La Trobe University, Melbourne, Australia; Fundação Getúlio Vargas: Fundacao Getulio Vargas, BRAZIL

## Abstract

The generation interval, defined as the time taken by an infector to create another infection from its time of infection, is a crucial quantity to be estimated during an infectious disease outbreak. It informs the timescale of the epidemic unfolding and makes it possible to calculate the basic reproductive ratio, which quantifies the transmission potential of an infection, from incidence data. While the intrinsic generation interval remains stable during an outbreak in the absence of interventions and behavioural changes, the generation intervals of successful infection events, ‘realised generation intervals’, change over time depending on the dynamics of the epidemic and how data are aggregated to define either the forward or the backward generation intervals. These time varying distributions are well understood for homogeneous, well-mixed populations, and can be used to infer the intrinsic generation interval distribution. For heterogeneous populations, the state-of-the-art method relies on the use of expensive network-based or agent-based simulations. We use the edge-based compartmental modelling framework to develop exact formulae for the generation time distribution of a Markovian SIR infection spreading on a heterogeneous contact network. These formulae are validated using stochastic outbreak simulations and relate backward and forward generation intervals with the intrinsic generation intervals. Finally, we use our results to demonstrate some previously unexplored biases in the estimation of the intrinsic generation times from the realised one, which could be caused by the incorrect assumptions about the network structure in the model and particularly the temporal structure of contacts.

## Author summary

When a new infectious disease outbreak starts, mathematical epidemiologists find themselves building or using mathematical models to understand its propagation, predict its future course, and recommend interventions to policy-makers. A key part of this process is the estimation of model parameters, including the generation intervals, i.e., the time taken by a primary case to infect a secondary case since its infection. Generation interval informs the duration of isolation and quarantine and provides a necessary input for the basic reproductive number estimation, which quantifies the transmission potential of the infection. However, the interpretation of the observed generation time is generally done under the assumption of a homogeneous, well-mixed population. Our work provides insight into the biases that might creep into estimates of parameters due to this assumption. We derive formulae that relate different types of commonly used generation intervals for a population with a heterogeneous contact network. These findings will inform infectious disease modellers on how the assumptions they make about the contact structure might bias the parameter estimation.

## 1 Introduction

Generation time, the time a parent takes to produce an offspring since its birth, is an important quantity of interest in ecology. The concept has been used extensively in epidemiology, where it refers to the time taken for a newly infected individual to infect someone else. Knowing this interval distribution is necessary for planning contact tracing, quarantine, and isolation in case of an outbreak [1]. It is also necessary to estimate ${ℛ}_{0}$, which quantifies the epidemic transmission potential, from the aggregated incidence of infections using the Wallinga-Lipsitch equation [2]. However, measuring this quantity and correctly interpreting it for outbreak analysis is not straightforward [3]. While an intrinsic generation interval can be defined, the observed generation interval, which is computed from available epidemiological data, is different from the intrinsic one.

The intrinsic generation interval is defined as the time distribution of infectious contacts made by a primary case, or, equivalently, the time distribution of generated infections when the population is fully susceptible and mixes randomly. In the case of a randomly mixing population, this is the distribution that is used in the Wallinga-Lipsitch equation. This definition of intrinsic generation intervals is consistent with previous definitions in the literature [3]. Their distribution is governed by the expected infectiousness as a function of time and the distribution of infectious duration. Infectiousness is a combination of biological, behavioural (e.g., use of mask or other protection measures) and environmental factors. Thus, these factors can affect the intrinsic generation intervals [4]. This definition implies that intrinsic generation intervals are independent of the contact structure of the population and do not depend on whether a transmission event (or infectious contact) leads to a successful infection/secondary case.

The observed or realised generation intervals, obtained by contact tracing data, are different from the intrinsic ones because of dynamical and population mixing effects, and also because of how generation intervals are defined and aggregated from available data. For instance, the forward generation interval distribution refers to the distribution of times it takes for a group of individuals infected at a given time to transmit the infection to others. On the other hand, the backward generation interval distribution takes the point of view of a cohort of infectees, infected at a given time, and is defined as the distribution of times to infect them [3].

Interventions or behaviours can affect the infectiousness and duration of infection, which in turn can affect the intrinsic generation intervals. Such effects have been documented in the case of SARS-Cov-2 [5]. In the absence of interventions and behavioural changes, one would expect the intrinsic generation intervals to remain the same throughout an outbreak. In contrast, forward and backward realised generation times vary due to the inherent dynamics of susceptible depletion and changes in incidence. The functional relationship between intrinsic and realised generation time was mathematically studied for a homogeneous randomly mixing population by Champredon and Dushoff within the renewal equation framework [3]. The mean backward generation time increases monotonically, underestimating the intrinsic generation interval early on and overestimating it later. This bias arises from the incidence dynamics. During the growth phase, recent infections dominate the pool of potential infectors, skewing generation times shorter; during the decline, older infections dominate, lengthening the generation intervals. On the other hand, the forward-looking formulation of generation time avoids this dynamic bias. Still, its mean value shrinks right before the time of peak prevalence [6,7]. One reason for this contraction is the high rate at which susceptibles are being depleted near the peak. An infector may generate multiple transmissions throughout their infectious period and, if the number of susceptibles remains the same, then the transmission events are all equally likely to contribute to the generation intervals. Conversely, if the availability of susceptibles shrinks significantly during the infectious period the later transmissions are less likely to lead to an infection, and longer transmission intervals are less likely to be sampled. An alternative mechanism to understand the generation time contraction is the competition between infectors racing to infect someone [6,8,9]. When many infectors compete to infect the same susceptible individual, only the transmission by the fastest infector counts as an infection event and contributes to the generation time statistics, causing the generation interval to contract. The study of these dynamical mechanisms is essential to design techniques to fit the intrinsic generation time from contact tracing data [10–12].

Although most studies rely on the homogeneous mixing assumption, contacts within households, schools, and workplaces are recurrent, heterogeneous, and spatially clustered [13–20]. Previous studies investigating clustering and recurrence showed that these enhance the competition between infectors and cause a stronger reduction of generation intervals — more than predicted by homogeneous-mixing models — which persists throughout the outbreak [9,13]. Despite these findings, the effect of the contact network on the generation time dynamics remains under-studied, especially from an analytical perspective. Here, we use the edge-based compartmental modelling framework to derive exact equations for realized generation intervals [21]. The framework allows us to systematically address the role of contact heterogeneity by comparing a homogeneous network with a heterogeneous network, and contact recurrence by comparing the cases in which contacts’ identities continuously change or are fixed. Our theoretical framework, supported by simulations, provides insights into the dynamics of generation intervals and highlights differences between network topologies. Most importantly, it points to potential sources of bias that were previously unexplored.

## 2 Methods

### 2.1 Champredon and Dushoff equations

Before introducing the formalism for generation-time distributions on networks, we briefly review the relationships between realised and intrinsic generation times, derived by Champredon and Dushoff equations for the special case of a homogeneous, well-mixed population [3]. All variables and functions introduced in this and the following sections are listed in Table 1. The Champredon and Dushoff [3] equations are the following:


$$\begin{array}{c} f_{H}(t,\tau )=\frac{g(\tau )S(t+\tau )}{\int_{0}^{\infty }g(x)S(t+x)\text{d}x}, \end{array}$$ (1a)

$$\begin{array}{c} b_{H}(t,\tau )=\frac{g(\tau )i(t-\tau )}{\int_{0}^{\infty }g(x)i(t-x)\text{d}x}. \end{array}$$ (1b)

**Table 1:** Table of variables and parameters.

Function/variable/parameter	Interpretation
(*S*, *I*, *R*)	Proportion of susceptible, infected and recovered individuals in the population
*i*(*t*)	Incidence at time *t*, or the rate at which new infections are created at time *t*
β	Transmissibility
γ	Rate of recovery from infection
θ(t)	Probability that a randomly chosen partner of a randomly chosen node has not transmitted to it yet
$p_{\ell }$	Probability that a node has degree *ℓ*
$\psi (x)=\sum p_{\ell }x^{\ell }$	Probability generating function
$\left(\pi_{S},\;\pi_{I},\;\pi_{R}\right)$	Probability of contact with a node in state (*S*, *I*, *R*), respectively, in an annealed network
$\left(\phi_{S},\;\phi_{I},\;\phi_{R}\right)$	Probability of contact with a node in state (*S*, *I*, *R*), respectively, in a quenched network
$g(\tau )=\gamma e^{-\gamma \tau }$	Density of intrinsic generation time distribution for a Markovian SIR dynamics
$\tilde{g}(\tau )=(\beta +\gamma )e^{-(\beta +\gamma )\tau }$	Density of pair-wise first transmission times for a Markovian SIR dynamics in a quenched population.
$\tilde{G}(\tau )=\int_{0}^{\tau }\tilde{g}(x)\text{d}x=e^{-(\beta +\gamma )\tau }$	Cumulative density of $\tilde{g}(\tau )$, defined above.
$f_{H}(t,\tau )$	Density of forward generation time distribution at time *t* in a well-mixing homogeneous population
$f_{A}(t,\tau )$	Density of forward generation time distribution at time *t* in an annealed network
$f_{Q}(t,\tau )$	Density of forward generation time distribution at time *t* in a quenched network
$b_{H}(t,\tau )$	Density of backward generation time distribution at time *t* in a well-mixing homogeneous population
$b_{A}(t,\tau )$	Density of backward generation time distribution at time *t* in an annealed network
$b_{Q}(t,\tau )$	Density of backward generation time distribution at time *t* in a quenched network
${ℛ}_{0}$	Basic reproduction number
${ℛ}_{f}^{A}$	Forward reproduction number in an annealed network
${ℛ}_{f}^{Q}$	Forward reproduction number in a quenched network
$u\overset{I\;}{\to }v$	Node *u* infects node *v*
u→v, u↛v	Node *u* transmits to node *v*, node *u* does not transmit to node *v*
ρ(event,t)	Rate at which the event $\overset{I\;}{\to }v$ or u→v occurs at time *t*

Equation (1a) relates the density of intrinsic generation times, g(τ), to the time-dependent density of forward generation times, $f_{H}(t,\tau )$, through the proportion of susceptibles in the population, *S*(*t*). On the other hand, equation (1b) relates the density of intrinsic generation times, g(τ), to the time-dependent density of backward generation times, $b_{H}(t,\tau )$, through the incidence, or the rate of new infections occurring, *i*(*t*). As we will discuss more in depth later in the text, the variables *S*(*t*) and *i*(*t*), in the above equations, represent the probabilities that a randomly selec*t*ed individual in the population enters into contact wi*t*h a suscep*t*ible or a newly infected individual, respectively. In the homogeneous mixing case, these are simply given by the probabilities that an individual is susceptible or newly infected, respectively.

Equations (1a) and (1b) assume a generic intrinsic generation interval distribution [3]. Throughout this manuscript, however, we focus on a simple model of infection history, the Markovian susceptible-infected-recovered (SIR). In a well-mixed and homogeneous population, the model has two parameters, the transmissibility, β and the rate of recovery, γ. Transmissibility is the rate at which an infected person transmits the infection to other individuals, while the rate of recovery informs the timing of recovery of an infected individual. In the Markovian SIR both parameters are constant in time, therefore the intrinsic generation interval distribution is the same as the recovery time distribution and has the exponential form $g(t)=\gamma e^{-\gamma t}$ [2].

We then review a property of equation (1b) that will be used later in the manuscript, when addressing the epidemiological implications of the effect of contact structure on the generation time estimation. In the initial stages of outbreaks, the infections grow exponentially, and so does the incidence. The density of backward generation times can be simplified in this stage. Using $i(t)\sim e^{\lambda t}$, equation (1b) becomes


$$\begin{array}{c} b_{H}^{\text{exp}}(\tau )=\frac{g(\tau )e^{-\lambda \tau }}{\int_{0}^{\infty }g(x)e^{-\lambda x}\text{d}x}. \end{array}$$ (2)

This density is independent of time *t*, indicating that when an epidemic is growing exponentially, the distribution of backward generation times is stable. In the case of a well-mixed Markovian SIR model, we have $g(t)=\gamma e^{-\gamma t}$, and λ=β−γ, which implies


$$\begin{array}{c} \begin{array}{c} b_{H}^{\text{exp}}(\tau )=(\lambda +\gamma )e^{-(\lambda +\gamma )\tau }=\beta e^{-\beta \tau },\\ \langle \tau \rangle_{\text{exp}}=\frac{1}{\beta }. \end{array} \end{array}$$ (3)

This equation suggests that if the backward generation times are observed during the exponential growth phase, then the transmissibility, β, can be estimated. In addition, the exponential growth rate, λ=β−γ, can be estimated from the time series of incidence. Thus, the parameter γ can be estimated, which gives us the distribution of intrinsic generation times. We will see that the stability of the backward generation intervals applies to all the models we discuss in this work and this fact will be used to get insights into the consequences of the contact structure in the *R*_0_ estimation.

### 2.2 Generalisation of the Champredon and Dushoff equations to a contact network

Homogeneous compartmental models make use of macroscopic variables such as the proportion of the population that is infected, susceptible, recovered, etc. The probability a random interaction with another individual is, e.g., with a newly infected individual or a susceptible one, is equal to the proportion of the population that is newly infected or susceptible, respectively. In a heterogeneous population, however, these contact probabilities no longer take the simple forms *S*(*t*) or *i*(*t*). Still, for annealed and quenched configuration model networks, these contact probabilities are well studied and can be derived within the Edge-Based Compartmental Modelling (EBCM) framework. In the next subsection, we will review the key concepts from EBCM and derive the contact probabilities, which enable the generalisation of the equations (1).

### 2.3 Edge-based compartmental modelling

This section gives a brief overview of the Edge-Based Compartmental Modelling (EBCM) framework [21] that is required for understanding the results in the next section. The EBCM framework can be used to solve the SIR model on configuration model networks. These networks are specified by the distribution of the degrees of the nodes (numbers of contacts), but are limited to a tree-like structure, i.e., they do not allow for clustering or higher-order structures.

Real-world networks have a temporal nature: some contacts of an individual are fleeting (fast changing) while others are static (long-lasting). The models used here will consider versions of configuration model networks for each type of contact. The first one is known as the annealed or mean field configuration model, where even though the number of contacts of an individual remains constant, the edges in the network rewire to new nodes constantly. The other is known as the quenched or static configuration model, where the edges are static.

We assume that both transmission and recovery follow Poisson processes. On a network, the transmission rate β is replaced by a per-contact transmission rate, so from this point onward β denotes the transmission rate per edge. As noted earlier, we consider Markovian transmission and recovery process, i.e., constant rates, throughout: each edge transmits at rate β, and infected individuals recover at rate γ. As seen before, the intrinsic generation interval is, therefore, exponential. The EBCM equations we use from [21] are derived for an SIR process, but the framework can be extended to other disease models—such as SEIR—as long as individuals cannot be reinfected. Consequently, non-exponential intrinsic generation-interval distributions can also be incorporated within this approach [22].

For both annealed and quenched networks, a standard compartmental modelling approach would divide the population up into sub-groups of homogeneous degree, resulting in at least as many differential equations as the number of sub-groups. The EBCM framework reduces such a large system of differential equations into two ordinary differential equations, which can be solved numerically with ease.

The EBCM framework makes use of probability-generating functions


$$\begin{array}{c} \psi (x)=\sum_{\ell }p_{\ell }\;x^{\ell }, \end{array}$$ (4)

where $p_{\ell }$ refers to the proportion or probability of nodes of degree class ℓ. A variable θ is defined to be the probability that a randomly chosen partnership of a randomly chosen node has not transmitted to it yet. If a node of degree *k* is susceptible, then none of its *k* partners have transmitted to it. The probability for the node to be susceptible (given its degree *k*) is $\theta^{k}$. The prevalence of susceptibles in the population can be computed using


$$\begin{array}{c} S=\sum_{k}p_{k}\theta^{k}=\psi (\theta ). \end{array}$$ (5)

The probability θ is the same for any node in a configuration model network. Let us consider two initially susceptible nodes *u*_0_ and *u*_1_. If *v*_0_ and *v*_1_ are neighbours of *u*_0_ and *u*_1_, they are chosen from the same distribution due to the configuration model assumption. Consequently, *u*_0_ and *u*_1_ are indistinguishable if we condition only on the fact that *v*_0_ and *v*_1_ are neighbours of *u*_0_ and *u*_1_, without having any other knowledge about *v*_0_ and *v*_1_. Thus, the probability *v*_0_ has not yet transmitted to *u*_0_ must be the same as the probability that *v*_1_ has not transmitted to *u*_1_.

### 2.4 Annealed network

As discussed above, in a more general case where the number of partners differs among individuals, the probability a random contact with another individual is with an infected individual is no longer equal to the proportion of the population that is infected. It is instead, the prevalence of half-edges in the population that are attached to an infected node. The variable *I*(*t*) (the proportion of the population that is infected) is generalised to a variable $\pi_{I}(t)$ which is the proportion of half-edges that connect to infected nodes. Similarly, we generalize $\pi_{S}(t)$ and $\pi_{R}(t)$ which are the prevalence of half-edges with susceptible and recovered nodes, respectively. We also define a new variable $\pi_{i}(t)$, which is the incidence of infected stubs, i.e., the instanteous rate at which half-edges with infected nodes are created by new infections.

We find that the probability that a random half-edge connects to a susceptible node is


$$\begin{array}{c} \pi_{S}=\frac{\sum_{k}kp_{k}\theta^{k}}{\sum_{k}kp_{k}}=\theta \frac{\psi^{\prime }(\theta )}{\psi^{\prime }(1)}. \end{array}$$

Knowing $\pi_{S}$ requires θ(t). Ref. [21] introduces the following differential equation for θ(t)


$$\begin{array}{c} \dot{\theta }=-\beta \theta +\beta \frac{\theta^{2}\psi^{\prime }(\theta )}{\psi^{\prime }(1)}-\gamma \theta \ln\theta , \end{array}$$

which can be solved assuming that at the start of the epidemic, the initially susceptible nodes have not received any transmissions, i.e., θ(0)≈1.

It is important to note that, within the same framework, it is also possible to compute the basic reproduction number and the initial exponential growth rate, which will be used later:


$$\begin{array}{c} {ℛ}_{0}=\frac{\beta }{\gamma }\frac{\langle K^{2}\rangle }{\langle K\rangle }, \end{array}$$ (6)

$$\begin{array}{c} \lambda =\beta \frac{\langle K^{2}\rangle }{\langle K\rangle }-\gamma , \end{array}$$ (7)

where $\langle K^{2}\rangle =\sum_{k}k^{2}p_{k}$ and $\langle K\rangle =\sum_{k}kp_{k}$ [21].

### 2.5 Quenched network

In the quenched case, the variable *S*(*t*) is generalised to a variable $\phi_{S}(t)$ which is the probability that a partner, *v,* of a randomly selected node, *u*, is susceptible under the assumption that *u* has not transmitted to *v*.


$$\begin{array}{c} \phi_{S}=\frac{\sum_{k}kp_{k}\theta^{k-1}}{\sum_{k}kp_{k}}=\frac{\psi^{\prime }(\theta )}{\psi^{\prime }(1)}. \end{array}$$

In this case, the following differential equation [21] can be written:


$$\begin{array}{c} \dot{\theta }=-\beta \theta +\beta \frac{\psi^{\prime }(\theta )}{\psi^{\prime }(1)}-\gamma (1-\theta ), \end{array}$$

which can be solved under the same initial conditions as before.

As in the annealed case, the basic reproduction number and the initial exponential growth rate can be computed, and they read:


$$\begin{array}{c} {ℛ}_{0}=\frac{\beta }{\beta +\gamma }\frac{\langle K^{2}-K\rangle }{\langle K\rangle }, \end{array}$$ (8)

$$\begin{array}{c} \lambda =\beta \frac{\langle K^{2}-2K\rangle }{\langle K\rangle }-\gamma , \end{array}$$ (9)

where $\langle K^{2}\rangle =\sum_{k}k^{2}p_{k}$ and $\langle K\rangle =\sum_{k}kp_{k}$ [21].

## 3 Results

### 3.1 Annealed network

In the Methods section, we have introduced the Champredon and Dushoff equation for a well-mixed homogeneous population, and we have reviewed how the probability of entering into contact with a susceptible individual changes from the homogeneous mixing to the contact network case. We have seen that within the EBCM formalism, and for an annealed network, such a probability is encoded in $\pi_{S}(t)$. Here, we combine these concepts to derive an equation for the forward generation interval distribution for an annealed network. The calculation details are reported in S1 File. We find that the forward generation interval distribution for an annealed configuration model network is


$$\begin{array}{c} f_{A}(t,\tau )=\frac{g(\tau )\pi_{S}(t+\tau )}{\int_{0}^{\infty }g(x)\pi_{S}(t+x)\text{d}x}. \end{array}$$ (10)

Turning our attention to the backward generation interval, the probability of contacting a newly infected node is not equivalent to the incidence of new infections, *i*(*t*), but instead, $\pi_{i}(t)$, the probability of entering into contact with a half-edge pointing to a newly infected node. The backward generation interval distribution is then (see S1 File for details)


$$\begin{array}{c} b_{A}(t,\tau )=\frac{g(\tau )\pi_{i}(t-\tau )}{\int_{0}^{\infty }g(x)\pi_{i}(t-x)\text{d}x}. \end{array}$$ (11)

It is important to observe that the two equations above are generalisations of the Champredon and Dushoff equations for forward generation time (1a) and backward generation time (1b), respectively, that reduce to those equations when the network is homogeneous. Indeed, we have seen that $\pi_{S}=\frac{\sum_{k}kp_{k}\theta^{k}}{\sum_{k}kp_{k}}$, where *p*_*k*_ is the probability mass function (PMF) of the degree distribution. If the support of the degree distribution consists of a single positive integer, or in other words, if all the nodes in the network have the same number of partners, then $\pi_{S}=S$. By replacing $\pi_{S}$ with *S*, equation (10) reduces to equation (1a). Similarly, when all the nodes have the same degree $\pi_{i}=i$, and thus equation (11) reduces to equation (1b).

Equation (2) shows that the backward generation interval distribution is stable in the exponential growth regime for a homogeneous population. This remains valid in the heterogeneous network case. The probability of newly infected half-edges, $\pi_{i}(t)$, has a linear dependence on the incidence of infections. Therefore, it has the same exponential growth rate as the outbreak. By plugging in $\pi_{i}(t)=\pi_{i}(0)e^{\lambda t}$ into the above formula, we have


$$\begin{array}{c} \begin{array}{c} b_{A}^{\text{exp}}(\tau )=(\lambda +\gamma )e^{-(\lambda +\gamma )\tau },\\ \langle \tau \rangle_{\text{exp}}=\frac{1}{\lambda +\gamma }. \end{array} \end{array}$$ (12)

It should be noted that the expression for $b_{A}^{\text{exp}}$ resembles $b_{H}^{\text{exp}}$ from equation (4), but differs as the initial growth rate for the annealed network is not β−γ, as it is in the homogeneous mixing case.

Similar to the forward generation interval, we define an effective reproduction number called the forward reproduction number, ${ℛ}_{f}$. It represents the expected number of infections created by an average infector who became infected at time *t* over its entire infectious period. For an annealed ne*t*work,


$$\begin{array}{c} {ℛ}_{f}^{A}(t)=\frac{\beta (\theta \psi^{\prime \prime }(\theta (t))+\psi^{\prime }(\theta (t)))}{\psi^{\prime }(\theta (t))}\int_{0}^{\infty }e^{-\gamma \tau }\pi_{S}(t+\tau )\text{d}\tau . \end{array}$$ (13)

If a reproduction number is calculated empirically through observation of transmission chains and corrected for censoring, we would expect it to match with the forward reproduction number. A detailed derivation for this expression is presented in S1 File.

### 3.2 Quenched network

We now derive the forward and backward generation interval equations for the case of a quenched network. As before, we provide the main calculations here and we refer to S1 File for additional details. In an annealed network, a node continuously breaks its edges and re-connects to new edges. So, if a node infects another node, it does not compete with itself when transmitting again. Conversely, in a quenched network, a node can compete with itself — after the first transmission, all later transmissions to the same neighbour have no effect. To account for the fact that only the first transmission along an edge leads to an infection, we introduce the pair-wise first transmission interval. Its distribution is related to the distribution of intrinsic generation intervals. The density of the pair-wise first transmission times can be calculated by considering a pair of connected nodes *u* and *v*, where *u* becomes infected, and *v* is susceptible at *t* = 0. Let us denote the event that *u* infects *v* (i.e., first transmission), using $u\overset{I\;}{\to }v$, and the event that *u* transmits to *v*, or attempts to infect *v*, using u→v. Since the infectiousness of *u* is constant in time and it recovers at a constant rate γ, the rate at which an infection event happens at time *t* is equal to the rate of transmission from *u* to *v* at time *t*, ρ(u→v,t), times the probability *t*hat *u* never transmitted to *v* before *t*


$$\begin{array}{c} \rho (u\overset{I\;}{\to }v,t)=\rho (u\to v,t)\times p(u↛v,t^{\prime }<t). \end{array}$$ (14)

For a Markovian SIR infection, transmissibility is the constant, β, but a transmission event also requires that *u* has not recovered. Therefore, $\rho (u\to v,t)=\beta e^{-\gamma t}$ and


$$\begin{array}{c} \rho (u\overset{I\;}{\to }v,t)=\beta e^{-\gamma t}\times e^{-\beta t}. \end{array}$$ (15)

Thus, the density of pair-wise first transmission times in a quenched network is obtained by normalising $\rho (u\overset{I\;}{\to }v,t)$.


$$\begin{array}{c} \tilde{g}(\tau )=(\beta +\gamma )e^{-(\beta +\gamma )\tau }. \end{array}$$ (16)

It should be noted that in the annealed case, the pair-wise first transmission interval matches the intrinsic generation interval. In the equations for the realised generation time, we will replace the density of intrinsic generation time g(τ) with the cumulative density of pair-wise first transmission times $\tilde{G}(\tau )=e^{-(\beta +\gamma )\tau }$. In addition, the probability that a stub points to a susceptible node – which was $\pi_{S}(t)$ in the annealed case – is now given by $\phi_{S}(t)$. The modified version of the equation in [3] then becomes


$$\begin{array}{c} f_{Q}(t,\tau )=\frac{\tilde{G}(\tau )\phi_{S}(t+\tau )}{\int_{0}^{\infty }\tilde{G}(x)\phi_{S}(t+x)\text{d}x}. \end{array}$$ (17)

In the backward case, the incidence of infections, *i*(*t*), is replaced with the average probability of incidence among the neighbours of a node, $\phi_{i}(t)$:


$$\begin{array}{c} b_{Q}(t,\tau )=\frac{\tilde{G}(\tau )\phi_{i}(t-\tau )}{\int_{0}^{\infty }\tilde{G}(x)\phi_{i}(t-x)\text{d}x}. \end{array}$$ (18)

From this equation, we again obtain that the backward generation intervals are stable in the exponential growth regime of the outbreak:


$$\begin{array}{c} \begin{array}{c} b_{Q}^{\text{exp}}(\tau )=(\lambda +\beta +\gamma )e^{-(\lambda +\beta +\gamma )\tau },\\ \langle \tau \rangle_{\text{exp}}=\frac{1}{\lambda +\beta +\gamma }. \end{array} \end{array}$$ (19)

In contrast to the annealed case, the equations for quenched networks do not immediately reduce to the equations derived by Champredon and Dushoff when the network is homogeneous. This is because of the effect of self-competition in quenched networks. However, for a homogeneous quenched network, in the limiting case that the transmission rate per edge tends to zero, β→0, and its degree becomes arbitrarily large, k→∞, while holding βk fixed, the probability that a node transmits along an edge is small, and the probability that it does so more than once is negligible. Then, in this limit, the epidemic on a quenched network behaves as if it is spreading on an annealed network. The considerations made above for the annealed case remain valid in this regime. Thus, the forward and backward generation time distribution equations for a quenched network converge to the homogeneous mixing equations when all nodes have the same arbitrarily large degree and the transmission rate per edge tends to zero.

Finally, the forward reproduction number is given by


$$\begin{array}{c} {ℛ}_{f}^{Q}(t)=\frac{\beta \psi^{\prime \prime }(\theta (t))}{\psi^{\prime }(\theta (t))}\int_{0}^{\infty }e^{-(\beta +\gamma )\tau }\phi_{S}(t+\tau )\text{d}\tau . \end{array}$$ (20)

A detailed derivation for the above derived equations is presented in S1 File.

### 3.3 Impact of network properties on the realised generation time

**Fig 1:**
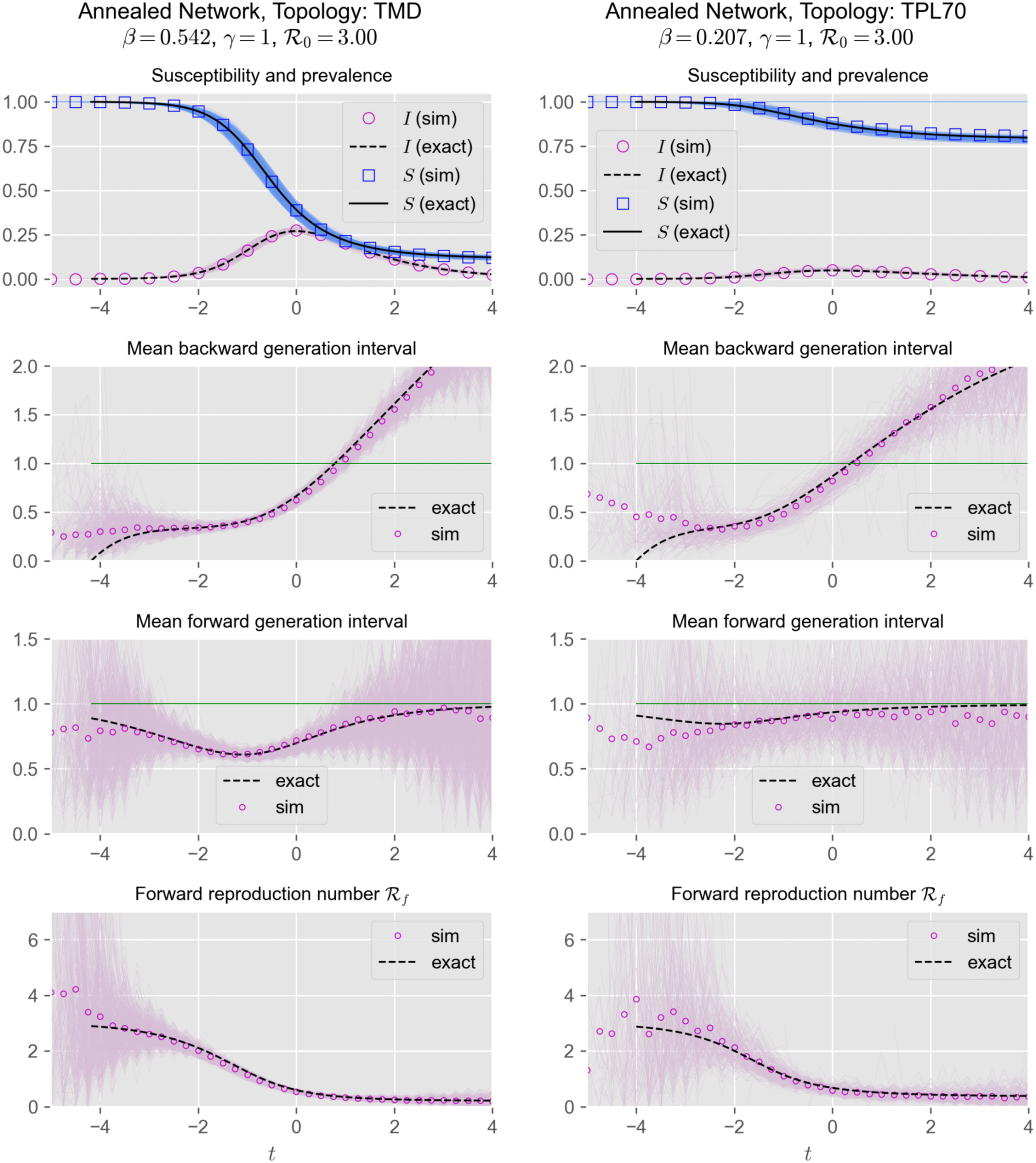
Comparison of realised generation intervals using stochastic simulations and the exact solution for annealed networks with two types of heterogeneous degree distributions. *Left: the degree of a node can be 3, 5, or 7 with equal probabilities. Right: degree distribution is a truncated power law with maximum degree of 70 and exponent of -2. We find a good match between theory and simulations for the realised generation intervals and the forward reproduction number. The time-series are centred such that the prevalence peaks at time zero. The thin lines show trajectories from each of the 500 simulations, while the plot with markers shows the average. At the start and towards the end of the epidemic, significant noise is observed and the simulation deviates from the theory*.

**Fig 2:**
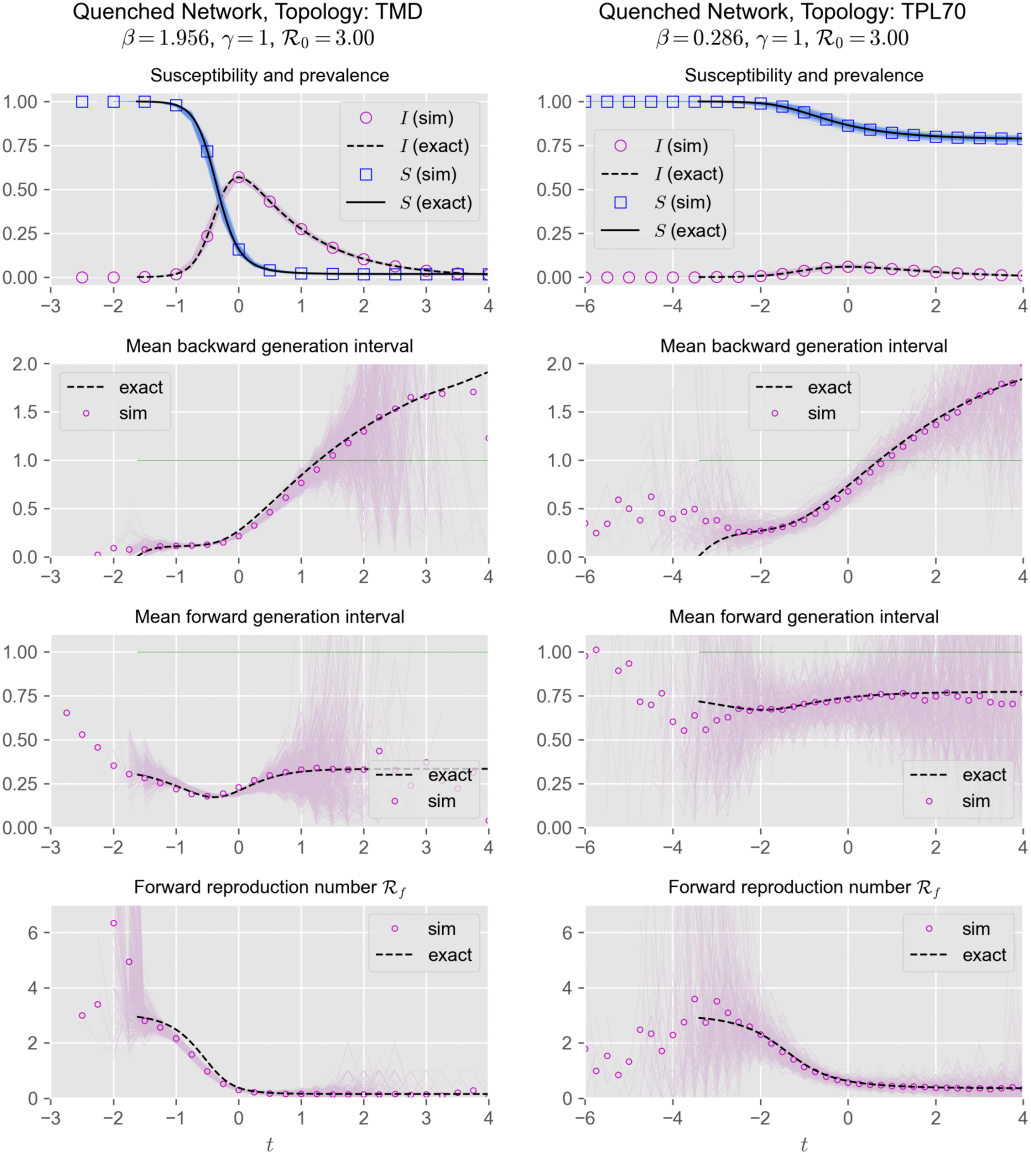
Comparison of realised generation intervals using stochastic simulations and the exact solution for quenched networks with two types of heterogeneous degree distributions. *Left: the degree of a node can be 3, 5, or 7 with equal probabilities. Right: degree distribution is a truncated power law with maximum degree of 70 and exponent of -2. We find a good match between theory and simulations for the realised generation intervals and the forward reproduction number. The time-series are centred such that the prevalence peaks at time zero. The thin lines show trajectories from each of the 500 simulations, while the plot with markers shows the average. At the start and towards the end of the epidemic, significant noise is observed and the simulation deviates from the theory for the truncated power law distribution*.

We validate the exact results using event-based simulations [23]. Figs 1 and 2 show the comparison between analytical solutions and simulations for the annealed and quenched case, respectively. In each figure, we plot the epidemic profile, the forward and backward generation times and the forward reproduction number. The figures show the comparison for two different topologies that we call TMD and TPL70. TMD refers to a tri-modal degree distribution and TPL70 refers to a truncated power law degree distribution (see figure captions for more details). Both networks are heterogeneous, but the TPL70 network is a case of extreme heterogeneity. The forward generation intervals in the annealed case show a contraction qualitatively similar to what is predicted by the homogenous mixing equations [3]. This is, however, less evident when the network is extremely heterogeneous. Although forward generation time varies substantially from one network to another, backwards generation time is quite robust to the network structure. In the quenched case, forward generation times shrink substantially due to the intensified competition among infectors.

We can further analyse the impact of the network features by numerically solving the derived analytical expressions for different scenarios. This is significantly inexpensive compared to stochastic numerical simulations. We conduct various numerical experiments to study the impact of heterogeneity on the mean of realised generation times. The results are summarised below and the associated figures are presented in S1 File (Figs A-C):

**Experiment (i)** Structure: Annealed TPL70 network and homogeneous network with the same mean degree. Disease parameters: Identical recovery rates (γ=1) and ${ℛ}_{0}$. Result: Heterogeneity reduces the contraction in the forward generation interval. However, this could possibly be explained by the lower peak (hence lesser competition) in the heterogeneity (See Fig A in S1 File).

**Experiment (ii)** Structure: Annealed TPL70 network and homogeneous network with same mean degree. Disease parameters: Identical recovery rate (γ=1). The transmission rate is selected such that the two epidemics have the same level of peak incidence of infections. Result: Heterogeneity increases the contraction in the forward generation interval, despite the same maximum incidence of infections. This can be explained by noting that the peak of $\pi_{i}(t)$ is higher than *i*(*t*). Furthermore, the backward generation intervals also differ significantly (See Fig B in S1 File).

**Experiment (iii)** Structure: Annealed TPL70 network. Method: Compare distribution of realised intervals assuming homogeneity [3] (i.e., using *i*(*t*) and *S*(*t*)) and not assuming homogeneity (i.e., using $\pi_{i}(t)$ and $\pi_{S}(t)$). Result: Using the net incidence of infections underestimates the contraction in forward generation interval. In contrast, the backward generation intervals are relatively unchanged (See Fig C in S1 File).

### 3.4 Implications for estimation of reproduction number

In this section, we will explore how errors could arise if disease parameters are inferred using existing methods to relate the observed generation interval and growth rate to the basic reproduction number.

If the intrinsic generation intervals and the exponential growth rate are known, the basic reproduction number can be estimated using the Wallinga-Lipsitch equation [2]. The growth rate can be estimated from the incidence at the start of the epidemic. However, the intrinsic generation intervals are not observed. Instead, we observe the realised generation intervals (forward or backward), which are influenced by the dynamics of the outbreak and the contact structure of the population.

So, to fit model parameters and infer the basic reproduction number from the growth rate and the observed generation intervals, it is crucial to recognise the distinction between the realised generation intervals and the intrinsic generation intervals. In principle, the exact expressions for realised generation interval distributions can be used to infer the intrinsic generation interval distribution, which can be used to then estimate ${ℛ}_{0}$ [3,9,11,12]. However, the Champredon and Dushoff’s equation and further developments rely on the homogeneous mixing assumption.

Our results from previous sections show that for a heterogeneous population, the conclusions will be incorrect if simple population aggregates, such as the prevalence of susceptibles or the incidence of infections, are used. We expect that the joint use of simple aggregate observations of an outbreak and realised generation intervals could bias the estimation of the basic reproduction number and the intrinsic generation intervals. To demonstrate this bias, we now look at an example case of outbreak analysis and two scenarios with different amounts of information available to the disease modeller.

#### 3.4.1 Example.

Let us consider an SIR pathogen that is causing an epidemic in two disconnected populations. In one population, the authorities have the capacity and resources to document the incidence of cases and also perform backward contact tracing (let us call it Population 1). In the other population, there is no capacity to perform contact tracing, but case incidence is documented (call it Population 2). Both populations are well-described by quenched and heterogeneous contact networks. This is a simplification, as any real-world network will have a mixture of short and long-time scale contacts. Furthermore, we assume that the populations have the same recovery rate ($\gamma_{1}=\gamma_{2}=\gamma$), while other parameters differ. The difference in the transmissibility for the two populations can be attributed to behavioural factors. The recovery rate, however, depends on biological factors and is independent of behaviour. Therefore, it is reasonable to assume that they are the same and is in fact a commonly used assumption.

First, we describe the quantities of interest in the ground reality from the correct model. Then, we consider cases where an infectious disease modeller has some information about the contact structure and see how their modelling assumptions can affect model inference in both populations. Detailed derivations of the mathematical equations used here can be found in S1 File section 1.3.

By applying the Wallinga-Lipsitch equation to the ground reality model (quenched network) and using equation (19),


$$\begin{array}{c} {ℛ}_{0}=1+\frac{\lambda }{\beta +\gamma }, \end{array}$$ (21)

$$\begin{array}{c} \langle \tau \rangle_{\text{exp}}=\frac{1}{\lambda +\beta +\gamma }, \end{array}$$ (22)

we can write the two basic reproduction numbers in terms of the observable quantities:


$$\begin{array}{c} {ℛ}_{0}^{1}=\frac{1}{1-\lambda_{1}\langle \tau_{1}\rangle_{\text{exp}}}, \end{array}$$ (23)

$$\begin{array}{c} {ℛ}_{0}^{2}=\frac{1}{1-\lambda_{2}\langle \tau_{2}\rangle_{\text{exp}}}, \end{array}$$ (24)

where the λ is the exponential growth rate estimated from incidence and $\langle \tau \rangle_{\text{exp}}$ is the mean backward generation interval during the exponential growth phase, estimated from the contact tracing data. Let us call these observable quantities. We can also write the rate parameter of the intrinsic generation time distribution as a function of the observable quantities and the degree distributions of the contact networks (see S1 File).

**Case A:** The modeller does not know anything about the contact structure and assumes a well-mixed homogeneous model. The model has two parameters, the transmission rate, β, and the recovery rate, γ, which also specifies the intrinsic generation intervals. Using this model, the estimators for population 1 are:


$$\begin{array}{c} {\hat{\gamma }}_{1}=\frac{1}{\langle \tau_{1}\rangle_{\text{exp}}}-\lambda_{1}, \end{array}$$ (25)

$$\begin{array}{c} \hat{{ℛ}_{0}^{1}}=\frac{1}{1-\lambda_{1}\langle \tau_{1}\rangle_{\text{exp}}}. \end{array}$$ (26)

Comparing these estimates to the ground reality (equations 7–10 of S1 File) tells us that the basic reproduction number is unbiased but ${\hat{\gamma }}_{1}$ is not as it does not properly account for the contact network properties – first and second moment of the degree distribution. More precisely,


$$\begin{array}{c} \begin{array}{c} {\hat{\gamma }}_{1}=\gamma_{1}+\beta_{1}=\frac{\lambda_{1}}{\kappa_{1}}+\gamma_{1}\frac{\kappa_{1}}{\kappa_{1}-1},\text{ where}\\ \kappa_{1}=\frac{\langle K_{1}^{2}-K_{1}\rangle }{\langle K_{1}\rangle }. \end{array} \end{array}$$ (27)

For population 2, nothing is known about the backward generation intervals, but the modeller has an estimate for the intrinsic generation intervals in population 1 and might see it fit to treat ${\hat{\gamma }}_{1}$ as an estimator for $\gamma_{2}$. This leads to an estimate of the reproduction number


$$\begin{array}{c} \hat{{ℛ}_{0}^{2}}=\frac{1+(\lambda_{2}-\lambda_{1})\langle \tau_{1}\rangle_{\text{exp}}}{1-\lambda_{1}\langle \tau_{1}\rangle_{\text{exp}}}\neq \frac{1}{1-\lambda_{2}\langle \tau_{2}\rangle_{\text{exp}}}, \end{array}$$ (28)

which is biased. We find that the bias in the estimator for population 2 is greater when the contact structure is different among the two populations. When the contact structure is identical, the estimator is unbiased when the growth rates are equal.

**Case B:** The modeller knows that the contacts are quenched and also has correct information of the average number of contacts $\langle K_{1}\rangle$ and $\langle K_{2}\rangle$ respectively. In the absence of more detailed information, they assume a homogeneous quenched network with degrees $k_{1}=\langle K_{1}\rangle$ and $k_{2}=\langle K_{2}\rangle$, respectively. The model has two other parameters, the transmission rate β and the recovery rate γ (which also specifies the intrinsic generation intervals). Using this model, the estimators for population 1 are:


$$\begin{array}{c} {\hat{\gamma }}_{1}=\frac{(k_{1}-2)(1/\langle \tau_{1}\rangle_{\text{exp}}-\lambda )-\lambda }{k_{1}-1}, \end{array}$$ (29)

$$\begin{array}{c} \hat{{ℛ}_{0}^{1}}=\frac{1}{1-\lambda_{1}\langle \tau_{1}\rangle_{\text{exp}}}. \end{array}$$ (30)

Comparison with the ground reality model shows that ${\hat{\gamma }}_{1}$ is biased, as calculation does not correctly account for the second moment of the degree distribution, while $\hat{{ℛ}_{0}^{1}}$ is not. As in the previous case, the modeller might see fit to treat ${\hat{\gamma }}_{1}$ as an estimator for $\gamma_{2}$. This leads to an estimate of the reproduction number for population 2


$$\begin{array}{c} \hat{{ℛ}_{0}^{2}}=\frac{\left(\lambda_{2}+{\hat{\gamma }}_{1})(k_{2}-1\right)}{\lambda_{2}+{\hat{\gamma }}_{1}(k_{2}-1)}. \end{array}$$

This estimator is biased, albeit behaves very differently than the biased estimator in Case A.

**Fig 3:**
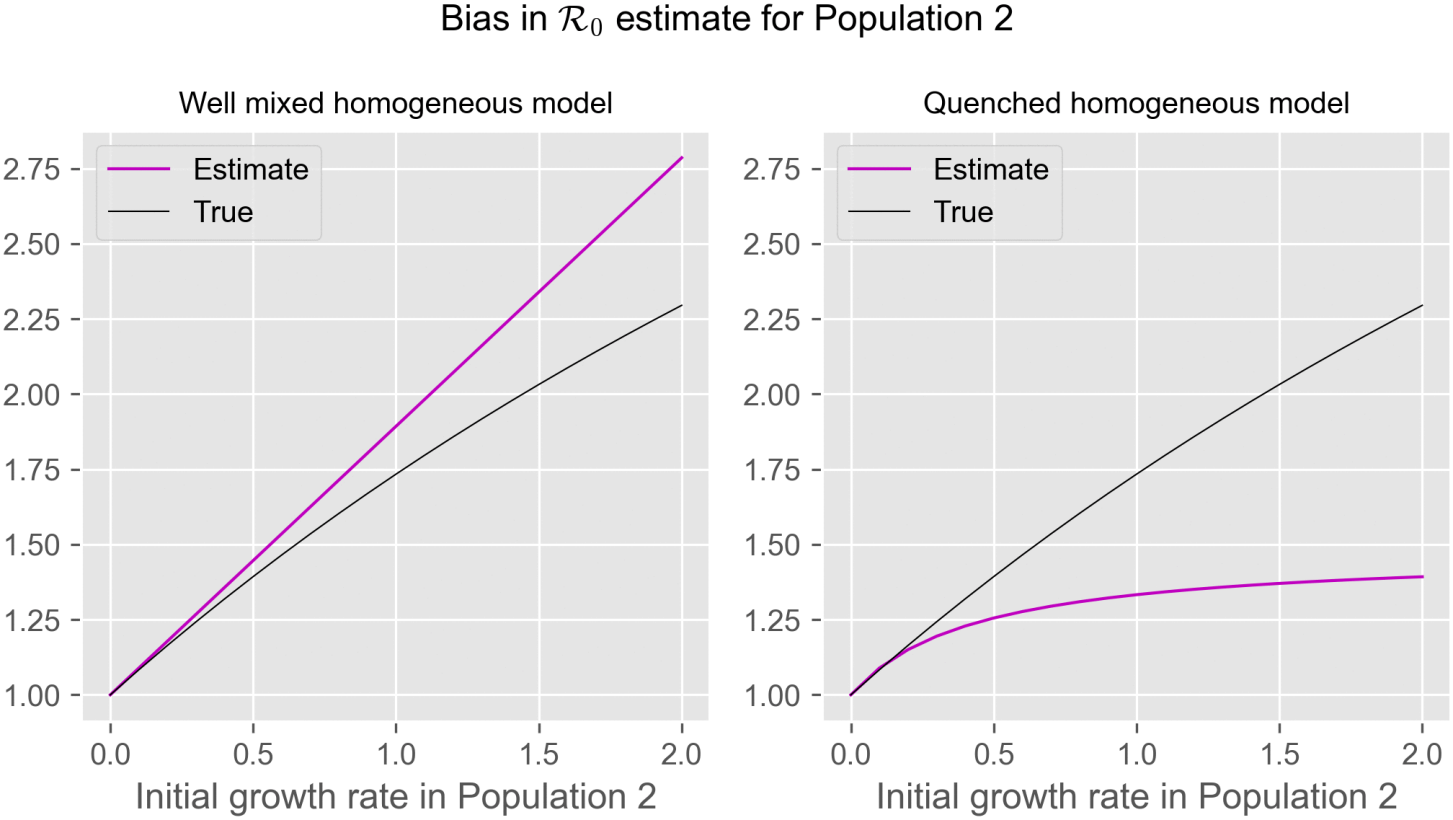
Bias in estimation of ${ℛ}_{0}$. *Comparison between the estimated and true value of the basic reproduction number in a heterogeneous quenched network when different homogeneous models are used and the intrinsic generation interval is estimated from a different population (see* 3.4.1 *for a detailed explanation)*.

In summary, we find that if the incidence and backward contact tracing is available in a population, then even incorrect assumptions about the contact structure can yield an unbiased estimator for the basic reproduction number, even though the estimator for the intrinsic generation interval is biased. Using this estimated intrinsic generation interval in order to estimate the basic reproduction number in another population leads to biased estimates. See Fig 3 for an illustration of the bias in $\hat{{ℛ}_{0}^{2}}$.

Paradoxically, case B displays a more severe bias despite the analysis drawing on more information. This emphasises that heterogeneity in contact activity is an important factor that should be monitored alongside the mean during an outbreak. For Population 1, a truncated power law degree distribution is used with an exponent of -2 and maximum degree of 70. Population 2 has a similar structure but with a maximum degree of 30. The growth rate in Population 1 is $\lambda_{1}=0.5$ and recovery rate is γ=1.

## 4 Discussion

In this article, we have generalised Champredon and Dushoff’s theory [3] for realised generation intervals in a homogeneous and well-mixed population to heterogeneous networks, using the EBCM framework. This framework allows incorporating degree heterogeneity in a tree-like network. In structured populations, realised generation intervals have been investigated using network and agent-based simulations [13,24]. These methods benefit from their ability to be modified quite easily – adding more coarse-grained structure or even including forms of structure beyond degree heterogeneity. However, they are slow and managing the noise reasonably would require a large number of simulations or a large population. In addition, they do not provide analytical insights. Some analytical considerations were provided by a few works [9,25]. Our exact expressions, validated using simulations, present an advance in our ability to predict generation intervals in simple heterogeneous contact structures and set up a foundation to obtain exact expressions for more complex heterogeneous networks.

Generation intervals are useful in estimating the basic reproduction number of an epidemic. Champredon and Dushoff showed how fitting the mean intrinsic generation interval to the mean of the realised generation intervals (backward) is incorrect [3]. They anticipate that heterogeneity may affect their results. We confirm this, determine the manner by which it affects the results and more importantly, through what mechanism. We found that the realised intervals depend not on simple aggregate measures like net prevalence of susceptibles or net incidence of infection, but on more involved measures such as the prevalence of half-edges in the network, which have a susceptible or a newly infected node attached to them. Measurement of such quantities requires higher resolution observation of the population, at the scale of groups which are homogeneous in the number of contacts. Such measurements would prove to be unrealistic, and thus the bias induced by heterogeneity might be hard to rectify in practice. Future work should explore in depth the dependence of the bias on the network and biological parameters to provide a reference for future outbreak analysis efforts. However, knowing network statistics and their variations from one population to another may be hard, as these may depend among the others on the specific socioeconomic, as well as epidemic and public health contexts [26,27].

We also use a series of hypothetical experiments to show that the estimates of intrinsic intervals (inferred using realised generation intervals) are sensitive to model assumptions. Despite this sensitivity, the estimates of ${ℛ}_{0}$ can be relied upon as long as the estimated intrinsic generation intervals are used in the same setting where they were estimated. If the estimated intrinsic intervals are used in a different setting (say an epidemic caused by the same pathogen in a different setting where the contact/network structure differs) then the estimates of ${ℛ}_{0}$ will be biased. This practice of applying generation interval estimates or related parameter estimates from one setting to an epidemic of the same pathogen in another setting is common [28–33]. Conventionally, the realised generation intervals are obtained through contact tracing, but recent advances in genomic epidemiology have offered alternative methods to estimate realised generation intervals [34]. These methodological advances would not address the biases highlighted here, as these arise from incorrect assumptions about the contact structure.

The forward reproduction number that was derived here is an effective reproduction number. Effective reproduction numbers are of two types: instantaneous or cohort-based (case reproduction number). The instantaneous reproduction number measures the transmission happening at certain time while the case reproduction number measures the transmission that is caused by a cohort of infectors who became infected at a certain time [35]. The forward reproduction number, ${ℛ}_{f}$, is equivalent to the case reproduction number [35,36].

A key assumption throughout this work is that the recovery from infection is a Poisson process, i.e., the intrinsic generation interval distribution is exponential. Real-world diseases do not follow this assumption for many reasons – the biological infectiousness can vary through the progression of an infection, and the rate of recovery does depend on the time since infection. However, the expressions for forward and backward generation intervals that we derived can be generalised to these more complex cases. Furthermore, we assumed that transmission and recovery rates do not change during the course of the epidemic, so neither does the intrinsic generation time. Intervention or spontaneous behavioural changes, such as hygiene measures or mask wearing, could affect these parameters. Lockdown or social restrictions, instead, would not alter the intrinsic generation time (under the definition adopted here) but would alter the contact network, thus affecting the realised generation time, the basic reproductive ratio and the incidence growth rate. Changes in serial interval were documented during the COVID-19 pandemic [5,37,38]. Accounting for these aspects within the network framework introduced here would be an important future research direction. Finally, real-world networks have many kinds of heterogeneities beyond the kind we have investigated here. They have clustering (triads that form a triangle), multiple layers and temporal variation. Here, we have only studied degree heterogeneity in two extreme cases of temporal variation – quenched (static partners) and annealed (fleeting contact with changing partners). In principle, a linear combination of the distributions derived here may be used to model contact networks with a more realistic temporal behaviour.

### Software

The attached Supporting Information provides details of the simulation methods, detailed derivations of the new formulae presented here and additional plots for the results section. The software used in this study is available at https://github.com/praty-k/gi-networks

## Supporting information

S1 File Supporting information: methods and results. (PDF)
