# Supplementary material for: Network structure induced bias in estimates of intrinsic generation times
Source: PLoS Comput Biol. 2026 May 12;22(5):e1014239. doi: 10.1371/journal.pcbi.1014239 (PMC13245868; doi:10.1371/journal.pcbi.1014239)
Supplement: Attachment — Submitted filename: review-PCOMPBIOL-D-25-01054.pdf [file pcbi.1014239.s002.pdf]

Review of PCOMPBIOL-D-25-0105: Network structure induced bias in estimates of intrinsic generation times

July 1, 2025

This paper will be an extremely useful contribution to the literature on generation intervals and R_0, which has been laser-focused (often implicitly) on mass-action models. The consideration of the annealed and quenched network cases is an innovative way to handle the generalized mass-action models and models based on configuration-model contact networks simultaneously. The discovery of new sources of bias in observing generation intervals is both theoretically interesting and has practical implications for public health, where generation intervals play a leading role in infectious disease epidemiology.

My primary concern the lack of a clear definition of the intrinsic generation interval distribution $g(\tau)$ that applies to both the annealed and quenched network cases. In Wallinga and Lipsitch (2007) and Champredon and Dushoff (2015), we get $g(t) = \gamma e^{-\gamma\tau}$ in the case where infectiousness is constant and the infectious period has an exponential distribution with rate γ. In the mass-action case, $g(\tau)$ is the rate of infectious contacts (i.e., contacts sufficient to infect a susceptible) times the probability of remaining infectious for at least τ time units and divided by R_0. This definition can probably be used for the annealed network case (which is essentially a mass-action model with additional heterogeneity possibly induced by the degree distribution), but I'm not sure that it will work in the quenched network case. The derivations are clearly correct for the simulations presented in Section 3.3, but these appear to have exponential(β) times to infectious contact and exponential(γ) infectious periods. Although this is a special case, it is sufficient to establish previously unrecognized sources of bias in the estimation of intrinsic generation interval distributions. Thus, this concern does not diminish the usefulness or novelty of the paper.

More detailed comments—most of them minor—are below.

Comments

1. (page 2) The Introduction is an outstanding overview of generation intervals. In the third-to-last sentence ("The framework allows us…"), using

parentheses instead of dashes might improve readability. In the last sentence, it might be better to say “that were previously unexplored” instead of “that were left unexplored” and the comma is not necessary.

2. (page 3, just before Section 2.1) Using N_i instead of something like p_i for the proportion of nodes with degree i is a bit disorienting—though the notation is clearly defined in the text. Also, it would help to provide a brief explanation of why the probability θ of no transmission should be the same for all edges in a configuration model network.

3. (pages 3-4) It would help to define β and γ explicitly and to state the assumptions from Ref. 18 that lead to the differential equations. In particular, it is relevant that an infectious node transmits infection at a constant rate β and recovers from infectiousness at a constant rate γ. This is probably what is meant by “transmission and recovery from infection are described by a Poisson process”, but I think explicitly specifying the distribution of time to infectious contact and time to recovery from infectiousness would be more helpful and provide definitions of β and γ. It would also help to explain the extent to which these assumptions might be hard-wired into the edge-based compartmental modeling framework.

4. (definition of intrinsic generation intervals) Intrinsic generation intervals need to be defined clearly for both the annealed and quenched networks Given the assumptions from Ref. 18, it is not clear to me that the differential equations are consistent with an intrinsic generation interval that is not exponential. Contact interval distributions can be defined consistently across network-based and mass-action models: see Section 2.7 in Kenah (*Biostatistics*, 2011). A similar approach might work here.

5. (page 4) For readers who are not physicists, it would be helpful to write the dx after the integrands. Ideally, the differential operator should be non-italicized and have a small space before it. The integrals are perfectly correct as written, but it was distracting.

6. (page 5, just before Section 3.1) It would help to note that a reproduction number calculated by observing transmission chains would need to be corrected for censoring (i.e., to account for secondary infections not yet caused by the individuals at the end of the chains) in order to match the forward reproduction number. Otherwise, you are up against the fact that a tree always has n nodes and $n - 1$ edges (or a forest of k trees always has n nodes and $n - k$ edges).

7. (page 5, Section 3.2) It's not clearly justified that any $g(\tau)$ can be replaced by the same $\tilde{G}(\tau) = e^{-(\beta+\gamma)\tau}$. The parameters β and γ need to be clearly defined. This relates to my earlier comments about the definition of $g(\tau)$ and the differential equations from Ref. 18.

8. (page 5, Section 3.3) The details of the simulations need to be more clearly described. It seems like they assume exponential(β) times to infectious

contact and exponential(γ) infectious periods. The derivations in Sections 3.1 and 3.2 are clearly borne out by the simulations, but I suspect that both rely on these assumptions—which were also made in Ref. 18. Even if I am right, this does not compromise the importance of the paper.

9. (Figures 1 and 2) In the top right panel of Figure 1, the legend should be shifted slightly upward to be farther away from the I curve. In the top two panels of Figures 1 and 2, the line showing the exact solutions for I are difficult to see.

10. (page 8, bottom) In the last paragraph, "sectionss" should be "sections".

11. (page 9, equations 1-5) It might look better if the subscripts for the mean backward generation intervals were not italicized: T_{exp} instead of T_{exp}.

12. (page 9, last paragraph) It is not clear to me why intrinsic generation intervals could not vary between subpopulations. Couldn't the infectiousness profile and infectious period distribution differ from one subpopulation to another? The justification for a single population-level intrinsic generation interval would then rely on some assumptions about mixing (which probably hold in the cases considered here). This relates to my previous comments about the need to define the intrinsic generation interval more clearly.

13. (page 9, bottom) The text after equation (5) should not be indented.

14. (Section 3.4) These examples of bias are very interesting, and it is also interesting that the bias can go in both directions. The transfer of generation interval distributions from one setting to another is quite common, so it is good to sound a note of caution.

15. (Figure 3) It might help to say "initial growth rate" instead of just "growth rate" in the x-axis label. Also, it would help to add a grid to the background of each panel.
