# Supplementary material for: Network structure induced bias in estimates of intrinsic generation times
Source: PLoS Comput Biol. 2026 May 12;22(5):e1014239. doi: 10.1371/journal.pcbi.1014239 (PMC13245868; doi:10.1371/journal.pcbi.1014239)
Supplement: Attachment — Submitted filename: Response to reviewers - PCB.pdf [file pcbi.1014239.s005.pdf]

Response to reviewer #1

This paper will be an extremely useful contribution to the literature on generation intervals and R_0, which has been laser-focused (often implicitly) on mass-action models. The consideration of the annealed and quenched network cases is an innovative way to handle the generalized mass-action models and models based on configuration-model contact networks simultaneously. The discovery of new sources of bias in observing generation intervals is both theoretically interesting and has practical implications for public health, where generation intervals play a leading role in infectious disease epidemiology.

My primary concern the lack of a clear definition of the intrinsic generation interval distribution $g(\tau)$ that applies to both the annealed and quenched network cases. In Wallinga and Lipsitch (2007) and Champredon and Dushoff (2015), we get $g(t) = \gamma e^{-\gamma\tau}$ in the case where infectiousness is constant and the infectious period has an exponential distribution with rate γ. In the mass-action case, $g(\tau)$ is the rate of infectious contacts (i.e., contacts sufficient to infect a susceptible) times the probability of remaining infectious for at least τ time units and divided by R_0. This definition can probably be used for the annealed network case (which is essentially a mass-action model with additional heterogeneity possibly induced by the degree distribution), but I'm not sure that it will work in the quenched network case. The derivations are clearly correct for the simulations presented in Section 3.3, but these appear to have exponential(β) times to infectious contact and exponential(γ) infectious periods. Although this is a special case, it is sufficient to establish previously unrecognized sources of bias in the estimation of intrinsic generation interval distributions. Thus, this concern does not diminish the usefulness or novelty of the paper.

We would like to thank the reviewer for their detailed and thorough feedback on our work. Based on the feedback, we have clarified our definition of intrinsic generation intervals (see second paragraph of the introduction). This definition is independent of the contact structure or the underlying network structure. Intrinsic generation times are concerned with transmission events (even those that do not lead to a new infection). A quenched network will certainly influence successful infection events but will not affect transmission events. Therefore, the intrinsic generation interval is given by $g(t) = \gamma e^{-\gamma\tau}$ for both quenched and annealed cases. However, we do introduce a new distribution called the pair-wise first transmission interval (section 3.2). These are intervals of transmission events that lead to an infection, along an edge. We had previously referred to this as the effective intrinsic generation time but have renamed it for clarity.

We would like to note that if one were to include contact structure in the definition of intrinsic generation intervals, then they would be equivalent to the pair-wise first transmission interval. We do not expect that this will change any of the results. We hope that this clarifies the concerns regarding the definition of intrinsic generation intervals.

More detailed comments—most of them minor—are below.

Comments

1. (page 2) The Introduction is an outstanding overview of generation intervals. In the third-to-last sentence ("The framework allows us…"), using parentheses instead of dashes might improve readability. In the last sentence, it might be better to say "that were previously unexplored" instead of "that were left unexplored" and the comma is not necessary.

   We have made changes according to this comment.

2. (page 3, just before Section 2.1) Using N_i instead of something like p_i for the proportion of nodes with degree i is a bit disorienting—though the notation is clearly defined in the text. Also, it would help to provide a brief explanation of why the probability θ of no transmission should be the same for all edges in a configuration model network.

   The variables have been changed from N_i to p_i. In addition, we have clarified the meaning of the "theta" variable in Section 2.3 (last paragraph).

3. (pages 3-4) It would help to define β and γ explicitly and to state the assumptions from Ref. 18 that lead to the differential equations. In particular, it is relevant that an infectious node transmits infection at a constant rate β and recovers from infectiousness at a constant rate γ. This is probably what is meant by "transmission and recovery from infection are described by a Poisson process", but I think explicitly specifying the distribution of time to infectious contact and time to recovery from infectiousness would be more helpful and provide definitions of β and γ. It would also help to explain the extent to which these assumptions might be hard-wired into the edge-based compartmental modeling framework.
   A table has been added to the methods section to define the functions, parameters and variables used in the paper. Section 2.1 has been added for additional clarity, with a paragraph dedicated to explaining the assumption of constant rates. Moreover, the comment about the assumptions of the EBCM equations is addressed in section 2.3.

4. (definition of intrinsic generation intervals) Intrinsic generation intervals need to be defined clearly for both the annealed and quenched networks Given the assumptions from Ref. 18, it is not clear to me that the differential equations are consistent with an intrinsic generation interval that is not exponential. Contact interval distributions can be defined consistently across network-based and mass-action models: see Section 2.7 in Kenah (*Biostatistics*, 2011). A similar approach might work here.
   In response to this comment and other comments, we have made changes throughout the paper to clarify which results are general and which ones depend on exponentially distributed intrinsic GIs. The reviewer is right that, the differential equations we use are

dependent on the assumption that the intrinsic generation interval is exponential. However, EBCM can be extended to other models, see second paragraph of section 2.3.

5. (page 4) For readers who are not physicists, it would be helpful to write the dx after the integrands. Ideally, the differential operator should be non-italicized and have a small space before it. The integrals are perfectly correct as written, but it was distracting. The differential operator has been edited to have non-italic “d”. The dx has also been written after the integrands.

6. (page 5, just before Section 3.1) It would help to note that a reproduction number calculated by observing transmission chains would need to be corrected for censoring (i.e., to account for secondary infections not yet caused by the individuals at the end of the chains) in order to match the forward reproduction number. Otherwise, you are up against the fact that a tree always has n nodes and $n-1$ edges (or a forest of k trees always has n nodes and $n-k$ edges). The sentence has been edited accordingly.

7. (page 5, Section 3.2) It’s not clearly justified that any $g(\tau)$ can be replaced by the same $\tilde{G}(\tau) = e^{-(\beta+\gamma)\tau}$. The parameters β and γ need to be clearly defined. This relates to my earlier comments about the definition of $g(\tau)$ and the differential equations from Ref. 18. Yes, that is correct. We have edited the paragraph to make it clear that it is only for exponential distributions. We also added a brief derivation of $\tilde{g}(\tau)$ to improve clarity.

8. (page 5, Section 3.3) The details of the simulations need to be more clearly described. It seems like they assume exponential(β) times to infectious contact and exponential(γ) infectious periods. The derivations in Sections 3.1 and 3.2 are clearly borne out by the simulations, but I suspect that both rely on these assumptions—which were also made in Ref. 18. Even if I am right, this does not compromise the importance of the paper. We have included more details about the simulation in the Supporting Information. Indeed, as the reviewer correctly note, simulations are based on the Markovian assumption, i.e. constant transmission and recovery rate which translate into exponential time to infection and recovery. In the Supporting Information, we make this assumption explicit. We also provide additional details on how simulations were carried out. We used the EoN package for simulating epidemics on networks. We have not gone into detail about the algorithms that EoN uses as they are well documented elsewhere, and we have cited those references. However, we describe how the data from EoN simulations was used to construct forward and backward distributions.

9. (Figures 1 and 2) In the top right panel of Figure 1, the legend should be shifted slightly upward to be farther away from the I curve. In the top two panels of Figures 1 and 2, the line showing the exact solutions for I are difficult to see. All figures in the main text have been redesigned for better clarity and presentation.

10. (page 8, bottom) In the last paragraph, “sectionss” should be “sections”. We have corrected this typo.

11. (page 9, equations 1-5) It might look better if the subscripts for the mean backward generation intervals were not italicized: T_{exp} instead of T_{exp}. The subscripts have been changed accordingly.

12. (page 9, last paragraph) It is not clear to me why intrinsic generation intervals could not vary between subpopulations. Couldn't the infectiousness profile and infectious period distribution differ from one subpopulation to another? The justification for a single population-level intrinsic generation interval would then rely on some assumptions about mixing (which probably hold in the cases considered here). This relates to my previous comments about the need to define the intrinsic generation interval more clearly. For the Markovian SIR case, the intrinsic generation interval, as we have defined it, depends only on the recovery rate, which is a biological property and should not depend on behaviour and contact structure. Then, the infectious period distribution will be the same for both populations. In addition, parameters such as the mean infectious period are often used across a wide variety of settings, so it is a common practice to use this assumption. For the quenched case, the pair-wise generation interval would differ between populations. We hope that the clarification of the definition of intrinsic generation intervals addresses the concerns in this comment as well.

13. (page 9, bottom) The text after equation (5) should not be indented. We have corrected this.

14. (Section 3.4) These examples of bias are very interesting, and it is also interesting that the bias can go in both directions. The transfer of generation interval distributions from one setting to another is quite common, so it is good to sound a note of caution. Thank you. We have noted this in the discussion in the following sentence: "This practice of applying generation interval estimates or related parameter estimates from one setting to an epidemic of the same pathogen in another setting is common" with multiple citations.

15. (Figure 3) It might help to say "initial growth rate" instead of just "growth rate" in the x-axis label. Also, it would help to add a grid to the background of each panel. We have changed the labels accordingly. All figures in the main text have been redesigned for better clarity, and a grid has been added.

Response to Reviewer #2:

This paper attacks an interesting problem in tracking infectious diseases – how measurable generation time distributions are biased by the underlying network structure. It is interesting and well developed but some results need to be better explained and justified. The discussion of the formulation and its consequences should be expanded to better capture the comparison (and in some cases convergence) to more familiar homogenous models as well as to clarify other practicalities.

We would like to thank the reviewer for their thorough feedback on our work.

Suggestions for improvements follow:

1. When presenting the equations in sections 2.1-2.2 the notation and meaning of \gamma and \beta has not yet been explained. The equation for R_f^A is introduced but its origin is unclear as no mention of a reference or supplementary section is provided. Analogous comments also hold for R_f^Q and its variables.
We have now paid attention to properly introduce all variables and explain the origin of the equations given in the main paper. Specifically, equations R_f^A and R_f^Q were derived in the Supporting Information. This is now clearly explained in the main paper. In addition, to enhance the readability of the mathematical treatment we have added a table in the methods section that introduces and defines the important functions, variables and symbols used in the paper.
2. When the epidemic grows exponentially both b_A^exp and b_Q^exp seem to maybe link to the generation time expected from a homogeneous model. Is this true? If it is, this may also help better expose the connections between these models and the SIR etc.
No they do not link to the generation time expected from a homogeneous model. The reason is because they depend on the growth rate, which is different in the different cases as it is affected by the heterogeneity in the population. Additional equations have been added throughout the paper to clarify the relationship between model parameters and the backward generation intervals during the exponential growth phase.

3. The use of the annealed and quenched networks is interesting and I enjoyed seeing the expansion of the Champredon and Dushoff equations. Given this comparison, I think it would also be good (and perhaps broaden readership) if the authors make explicit any conditions under which both network types collapse to the homogenous form, leading to the recovery of Champredon and Dushoff equations.
In the annealed case, if the network is homogeneous (all nodes have the same degree) then we recover the homogeneous case. In the quenched case, we need some more conditions to recover the homogeneous case – the degree needs to become very large, and the transmissibility needs to approach zero for the network to behave like an annealed homogeneous network. We have added these explanations in the results section.

4. The authors highlight that if the population is heterogeneous then using aggregates like the susceptible count will bias estimation of an R0 or an equivalent statistic. This is part of a broader discussion about what is measurable (e.g., intrinsic generation times are not usually observable). Continuing in that line, it would be useful to explain how measurable the

alternatives such as the number of susceptible links are and whether if it may be that the bias from using susceptibles is hard to overcome practically.
The reviewer is right that quantities such as number of susceptible or infected stubs are hard to measure practically and thus these biases are hard to overcome. We have added a sentence in the discussion to address this comment.

5. The authors comment that the intrinsic generation time remains constant throughout an epidemic. Is this true? There have been works discussing changes in the generation time due to interventions and trying to account for any biases this may introduce into estimates of R0/Rt [1,2]. Empirically there has also been investigation via the serial interval (which is of course not the same as the generation time but often used as an approximation) [3] but does raise the question about changing generation times. The authors should discuss these papers (and any I missed that also talk about the wider context of generation times) and, if the intrinsic generation time changes, what impact it would have on their results, formulae and derived R0/Rt like quantities.

In our work we have assumed that the intrinsic generation time does not depend on the network structure. We have now clearly specified that in the Introduction. Therefore, interventions which affect the contact network, e.g. lockdown, alter the realised generation time, but not the intrinsic one. Other kinds of interventions and behaviours, such as hygiene measures or wearing masks, can change the infectivity profile and thus affect the intrinsic generation times. An assumption we have made in our paper is that the transmissibility 'beta' is constant in time. Therefore, intrinsic generation times stay constant. This assumption implies that we have assumed that the behaviour of individuals does not change. The last paragraph of discussion has been edited to address this comment. Assumptions about constant behaviour have also been made clear in the introduction and methods.

We have cited the papers and discussed the papers suggested by the reviewer

6. Not enough context or explanation is provided for the equations in the case studies. From where are they derived or taken? This requires the reader to have too much background knowledge to fully grasp what is happening. Please explain in more detail there.

A detailed derivation of the equations in the case studies is provided in the Supporting Information. We have added a reference to the Supporting Information in the main text.

7. In Fig 1-2 no expression is given for how R0 relates to \beta and \gamma. Further, while I

note a GitHub link is provided, the simulations need to be better detailed. Additionally, no information is provided about the other statistics of the generation time (and in fact no distributions are plotted). This would be useful together with some comments about how (as these network models are heterogeneous and stochastic) representative and useful a deterministic estimate (e.g., of R0) will be in these settings.
Expressions for R0 for both annealed and quenched models have now been added to the methods section. As the size of the network becomes very large, we would expect the average of the stochastic epidemic to match with the deterministic models.

Plots of the distributions of the realised generation intervals using both exact and simulation methods have been added to the Supporting Information (Figures S4 and S5).

We have also added details of the simulation methods to the Supporting Information (section simulation methods).

8. As a key result of this work is not just the exact expressions but highlighting the bias from using just susceptible counts or incidence, the work would benefit from a figure of further simulation/explanation showing what would go wrong if we did use the naïve aggregate.
Experiment (iii) in Section 3.3, explores what happens when the naïve aggregates are used. Figure S3 in the Supporting Information shows the results of such a simulation.

9. Seems strange to use R to refer to severity in the abstract. Perhaps transmissibility?
The wording has been changed to make it more accurate.

10. There have been relatively recent studies [4] trying to get at serial intervals via genomic data. While not essential for the paper, it would be interesting to know if there is potential to overcome some of the biases the authors reveal with such data.

We have added a brief discussion about this paper in the third paragraph of the Discussion section. The biases that we have reported here do not depend on how the generation times of an epidemic were obtained, but depend on the assumption made regarding the contact structure.

References:
1. https://royalsocietypublishing.org/doi/10.1098/rsif.2022.0128
2. https://royalsocietypublishing.org/doi/10.1098/rspb.2023.1664
3. https://www.science.org/doi/10.1126/science.abc9004
4. https://www.nature.com/articles/s41467-023-40544-y
