# Supplementary material for: Network structure induced bias in estimates of intrinsic generation times
Source: PLoS Comput Biol. 2026 May 12;22(5):e1014239. doi: 10.1371/journal.pcbi.1014239 (PMC13245868; doi:10.1371/journal.pcbi.1014239)
Supplement: Peer review history

# Peer review: Decision Letter 0

**DOI:** 10.1371/journal.pcbi.1014239.r001

**Authors:** Denise Kühnert

7 Oct 2025

PCOMPBIOL-D-25-01054

Network structure induced bias in estimates of intrinsic generation times

PLOS Computational Biology

Dear Dr. Poletto,

Thank you for submitting your manuscript to PLOS Computational Biology. After careful consideration, we feel that it has merit but does not fully meet PLOS Computational Biology's publication criteria as it currently stands. Therefore, we invite you to submit a revised version of the manuscript that addresses the points raised during the review process.

Please submit your revised manuscript within 30 days Dec 07 2025 11:59PM. If you will need more time than this to complete your revisions, please reply to this message or contact the journal office at ploscompbiol@plos.org. When you're ready to submit your revision, log on to https://www.editorialmanager.com/pcompbiol/ and select the 'Submissions Needing Revision' folder to locate your manuscript file.

Please include the following items when submitting your revised manuscript:

* A rebuttal letter that responds to each point raised by the editor and reviewer(s). You should upload this letter as a separate file labeled 'Response to Reviewers'. This file does not need to include responses to formatting updates and technical items listed in the 'Journal Requirements' section below.

* A marked-up copy of your manuscript that highlights changes made to the original version. You should upload this as a separate file labeled 'Revised Manuscript with Track Changes'.

* An unmarked version of your revised paper without tracked changes. You should upload this as a separate file labeled 'Manuscript'.

If you would like to make changes to your financial disclosure, competing interests statement, or data availability statement, please make these updates within the submission form at the time of resubmission. Guidelines for resubmitting your figure files are available below the reviewer comments at the end of this letter.

We look forward to receiving your revised manuscript.

Kind regards,

Claudio José Struchiner, M.D., Sc.D.

Academic Editor

PLOS Computational Biology

Denise Kühnert

Section Editor

PLOS Computational Biology

**Additional Editor Comments:**

Both reviewers recognize this work as an important contribution to the field, and they offer constructive comments that will improve the manuscript.

**Journal Requirements:**

If the reviewer comments include a recommendation to cite specific previously published works, please review and evaluate these publications to determine whether they are relevant and should be cited. There is no requirement to cite these works unless the editor has indicated otherwise.

1) Please ensure that the CRediT author contributions listed for every co-author are completed accurately and in full.

At this stage, the following Authors/Authors require contributions: Pratyush Kumar Kollepara, Chiara Poletto, and Joel C. Miller. Please ensure that the full contributions of each author are acknowledged in the "Add/Edit/Remove Authors" section of our submission form.

The list of CRediT author contributions may be found here: https://journals.plos.org/ploscompbiol/s/authorship#loc-author-contributions

2) We ask that a manuscript source file is provided at Revision. Please upload your manuscript file as a .doc, .docx, .rtf or .tex. If you are providing a .tex file, please upload it under the item type u2018LaTeX Source Fileu2019 and leave your .pdf version as the item type u2018Manuscriptu2019.

3) Please provide an Author Summary. This should appear in your manuscript between the Abstract (if applicable) and the Introduction, and should be 150-200 words long. The aim should be to make your findings accessible to a wide audience that includes both scientists and non-scientists. Sample summaries can be found on our website under Submission Guidelines:

https://journals.plos.org/ploscompbiol/s/submission-guidelines#loc-parts-of-a-submission

4) Please upload all main figures as separate Figure files in .tif or .eps format. For more information about how to convert and format your figure files please see our guidelines:

https://journals.plos.org/ploscompbiol/s/figures

5) We have noticed that you have uploaded Supporting Information files, but you have not included a list of legends. Please add a full list of legends for your Supporting Information files after the references list.

6) Please amend your detailed Financial Disclosure statement. This is published with the article. It must therefore be completed in full sentences and contain the exact wording you wish to be published.

1) Please clarify all sources of financial support for your study. List the grants, grant numbers, and organizations that funded your study, including funding received from your institution. Please note that suppliers of material support, including research materials, should be recognized in the Acknowledgements section rather than in the Financial Disclosure

2) State what role the funders took in the study. If the funders had no role in your study, please state: "The funders had no role in study design, data collection and analysis, decision to publish, or preparation of the manuscript."

3) If any authors received a salary from any of your funders, please state which authors and which funders..

If you did not receive any funding for this study, please simply state: u201cThe authors received no specific funding for this work.u201d

7) Your current Financial Disclosure states, "The author(s) received no specific funding for this work.".

However, your funding information on the submission form indicates receiving funds .

Please indicate by return email the full and correct funding information for your study and confirm the order in which funding contributions should appear. Please be sure to indicate whether the funders played any role in the study design, data collection and analysis, decision to publish, or preparation of the manuscript.

**Reviewers' comments:**

Reviewer's Responses to Questions

**Comments to the Authors:**

**Please note here if the review is uploaded as an attachment.**

Reviewer #1: Please see attached PDF.

Reviewer #2: This paper attacks an interesting problem in tracking infectious diseases – how measurable generation time distributions are biased by the underlying network structure. It is interesting and well developed but some results need to be better explained and justified. The discussion of the formulation and its consequences should be expanded to better capture the comparison (and in some cases convergence) to more familiar homogenous models as well as to clarify other practicalities. Suggestions for improvements follow:

1. When presenting the equations in sections 2.1-2.2 the notation and meaning of \gamma and \beta has not yet been explained. The equation for R_f^A is introduced but its origin is unclear as no mention of a reference or supplementary section is provided. Analogous comments also hold for R_f^Q and its variables.

2. When the epidemic grows exponentially both b_A^exp and b_Q^exp seem to maybe link to the generation time expected from a homogeneous model. Is this true? If it is, this may also help better expose the connections between these models and the SIR etc.

3. The use of the annealed and quenched networks is interesting and I enjoyed seeing the expansion of the Champredon and Dushoff equations. Given this comparison, I think it would also be good (and perhaps broaden readership) if the authors make explicit any conditions under which both network types collapse to the homogenous form, leading to the recovery of Champredon and Dushoff equations.

4. The authors highlight that if the population is heterogeneous then using aggregates like the susceptible count will bias estimation of an R0 or an equivalent statistic. This is part of a broader discussion about what is measurable (e.g., intrinsic generation times are not usually observable). Continuing in that line, it would be useful to explain how measurable the alternatives such as the number of susceptible links are and whether if it may be that the bias from using susceptibles is hard to overcome practically.

5. The authors comment that the intrinsic generation time remains constant throughout an epidemic. Is this true? There have been works discussing changes in the generation time due to interventions and trying to account for any biases this may introduce into estimates of R0/Rt [1,2]. Empirically there has also been investigation via the serial interval (which is of course not the same as the generation time but often used as an approximation) [3] but does raise the question about changing generation times. The authors should discuss these papers (and any I missed that also talk about the wider context of generation times) and, if the intrinsic generation time changes, what impact it would have on their results, formulae and derived R0/Rt like quantities.

6. Not enough context or explanation is provided for the equations in the case studies. From where are they derived or taken? This requires the reader to have too much background knowledge to fully grasp what is happening. Please explain in more detail there.

7. In Fig 1-2 no expression is given for how R0 relates to \beta and \gamma. Further, while I note a GitHub link is provided, the simulations need to be better detailed. Additionally, no information is provided about the other statistics of the generation time (and in fact no distributions are plotted). This would be useful together with some comments about how (as these network models are heterogeneous and stochastic) representative and useful a deterministic estimate (e.g., of R0) will be in these settings.

8. As a key result of this work is not just the exact expressions but highlighting the bias from using just susceptible counts or incidence, the work would benefit from a figure of further simulation/explanation showing what would go wrong if we did use the naïve aggregate.

9. Seems strange to use R to refer to severity in the abstract. Perhaps transmissibility?

10. There have been relatively recent studies [4] trying to get at serial intervals via genomic data. While not essential for the paper, it would be interesting to know if there is potential to overcome some of the biases the authors reveal with such data.

References:

1. https://royalsocietypublishing.org/doi/10.1098/rsif.2022.0128

2. https://royalsocietypublishing.org/doi/10.1098/rspb.2023.1664

3. https://www.science.org/doi/10.1126/science.abc9004

4. https://www.nature.com/articles/s41467-023-40544-y

**********

**Have the authors made all data and (if applicable) computational code underlying the findings in their manuscript fully available?**

The PLOS Data policy requires authors to make all data and code underlying the findings described in their manuscript fully available without restriction, with rare exception (please refer to the Data Availability Statement in the manuscript PDF file). The data and code should be provided as part of the manuscript or its supporting information, or deposited to a public repository. For example, in addition to summary statistics, the data points behind means, medians and variance measures should be available. If there are restrictions on publicly sharing data or code —e.g. participant privacy or use of data from a third party—those must be specified.

Reviewer #1: Yes

Reviewer #2: Yes

**********

PLOS authors have the option to publish the peer review history of their article (what does this mean?). If published, this will include your full peer review and any attached files.

If you choose “no”, your identity will remain anonymous but your review may still be made public.

**Do you want your identity to be public for this peer review?** For information about this choice, including consent withdrawal, please see our Privacy Policy.

Reviewer #1: No

Reviewer #2: No

[NOTE: If reviewer comments were submitted as an attachment file, they will be attached to this email and accessible via the submission site. Please log into your account, locate the manuscript record, and check for the action link "View Attachments". If this link does not appear, there are no attachment files.]

**Figure resubmission:**

-->While revising your submission, we strongly recommend that you use PLOS’s NAAS tool (https://ngplosjournals.pagemajik.ai/artanalysis) to test your figure files. NAAS can convert your figure files to the TIFF file type and meet basic requirements (such as print size, resolution), or provide you with a report on issues that do not meet our requirements and that NAAS cannot fix.-->

After uploading your figures to PLOS’s NAAS tool - https://ngplosjournals.pagemajik.ai/artanalysis, NAAS will process the files provided and display the results in the "Uploaded Files" section of the page as the processing is complete. If the uploaded figures meet our requirements (or NAAS is able to fix the files to meet our requirements), the figure will be marked as "fixed" above. If NAAS is unable to fix the files, a red "failed" label will appear above. When NAAS has confirmed that the figure files meet our requirements, please download the file via the download option, and include these NAAS processed figure files when submitting your revised manuscript.

**Reproducibility:**

To enhance the reproducibility of your results, we recommend that authors of applicable studies deposit laboratory protocols in protocols.io, where a protocol can be assigned its own identifier (DOI) such that it can be cited independently in the future. Additionally, PLOS ONE offers an option to publish peer-reviewed clinical study protocols. Read more information on sharing protocols at https://plos.org/protocols?utm_medium=editorial-email&utm_source=authorletters&utm_campaign=protocols

Attachment Submitted filename: review-PCOMPBIOL-D-25-01054.pdf

Attachment Submitted filename: review-PCOMPBIOL-D-25-01054.pdf

# Author comment: Author response to Decision Letter 1

**DOI:** 10.1371/journal.pcbi.1014239.r002

9 Apr 2026

Attachment Submitted filename: Response to reviewers - PCB.pdf

# Editor report: Decision Letter 1

**DOI:** 10.1371/journal.pcbi.1014239.r003

**Authors:** Claudio Struchiner

14 Apr 2026

Dear Dr. Poletto,

We are pleased to inform you that your manuscript 'Network structure induced bias in estimates of intrinsic generation times' has been provisionally accepted for publication in PLOS Computational Biology.

Before your manuscript can be formally accepted you will need to complete some formatting changes, which you will receive in a follow up email. A member of our team will be in touch with a set of requests.

Please note that your manuscript will not be scheduled for publication until you have made the required changes, so a swift response is appreciated.

IMPORTANT: The editorial review process is now complete. PLOS will only permit corrections to spelling, formatting or significant scientific errors from this point onwards. Requests for major changes, or any which affect the scientific understanding of your work, will cause delays to the publication date of your manuscript.

Should you, your institution's press office or the journal office choose to press release your paper, you will automatically be opted out of early publication. We ask that you notify us now if you or your institution is planning to press release the article. All press must be co-ordinated with PLOS.

Thank you again for supporting Open Access publishing; we are looking forward to publishing your work in PLOS Computational Biology.

Best regards,

Claudio José Struchiner, M.D., Sc.D.

Academic Editor

PLOS Computational Biology

Denise Kühnert

Section Editor

PLOS Computational Biology

***********************************************************

# Editor report: Acceptance letter

**DOI:** 10.1371/journal.pcbi.1014239.r004

**Authors:** Claudio Struchiner

PCOMPBIOL-D-25-01054R1

Network structure induced bias in estimates of intrinsic generation times

Dear Dr Poletto,

I am pleased to inform you that your manuscript has been formally accepted for publication in PLOS Computational Biology. Your manuscript is now with our production department and you will be notified of the publication date in due course.

The corresponding author will soon be receiving a typeset proof for review, to ensure errors have not been introduced during production. Please review the PDF proof of your manuscript carefully, as this is the last chance to correct any errors. Please note that major changes, or those which affect the scientific understanding of the work, will likely cause delays to the publication date of your manuscript.

Soon after your final files are uploaded, unless you have opted out, the early version of your manuscript will be published online. The date of the early version will be your article's publication date. The final article will be published to the same URL, and all versions of the paper will be accessible to readers.

For Research, Software, and Methods articles, you will receive an invoice from PLOS for your publication fee after your manuscript has reached the completed accept phase. If you receive an email requesting payment before acceptance or for any other service, this may be a phishing scheme. Learn how to identify phishing emails and protect your accounts at https://explore.plos.org/phishing.

Thank you again for supporting PLOS Computational Biology and open-access publishing. We are looking forward to publishing your work!

With kind regards,

Anita Estes

PLOS Computational Biology | Carlyle House, Carlyle Road, Cambridge CB4 3DN | United Kingdom ploscompbiol@plos.org | Phone +44 (0) 1223-442824 | ploscompbiol.org | @PLOSCompBiol
